# Pathways for Strengthening Lived Experience Leadership for Transformative Systems Change: Reflections on Research and Collective Change Strategies

**DOI:** 10.1111/hex.70048

**Published:** 2024-10-03

**Authors:** Mark Loughhead, Ellie Hodges, Heather McIntyre, Nicholas Procter, Anne Barbara, Brooke Bickley, Lee Martinez, Leticia Albrecht, Lisa Huber

**Affiliations:** ^1^ Mental Health and Suicide Prevention Research and Education Group University of South Australia Adelaide South Australia Australia; ^2^ South Australia Lived Experience Leadership & Advocacy Network Adelaide South Australia Australia; ^3^ Department of Health and Wellbeing – South Australia Adelaide South Australia Australia

**Keywords:** collective impact, consumer leadership, health service, lived experience leadership, participatory action research, systems change

## Abstract

**Introduction:**

The Activating Lived Experience Leadership (ALEL) project was a South Australian participatory action research project that aimed to improve the ways lived experience is recognised, valued and integrated across mental health and social sector systems. ALEL was completed during 2019–2021, where it engaged 182 participants in generating community action and research knowledge.

**Objective:**

Our paper discusses the project's processes of building a collective partnership among lived experience leaders and other leaders from within the sector, so that the actions and strategies identified through research could be implemented by systems‐level impact. We describe the collaborative process and key learnings that resulted in eight key action areas for transformative systems change in South Australia.

**Methods:**

The project invited a diverse range of self‐identified lived experience and other leaders to be involved in a PAR process featuring formal qualitative research (focus groups, surveys and interviews) as well as community development activities (leaders' summit meetings, consultations, training and community of practice meetings). These processes were used to help us describe the purpose, achievements and potential of lived experience leadership. Project priorities and systems‐level analysis was also undertaken with lived experience sector leaders and project advisors across two leaders' summit meetings, integrating research outcomes with sector planning to define high‐level actions and a vision for transformational change.

**Results:**

Participatory action research as informed by systems change and collective impact strategies assisted the project to generate detailed findings about the experiences and complexities of lived experience leadership, and collective responses of how systems could better support, be accountable to and leverage lived experience perspectives, experience and peer‐work approaches.

**Conclusion:**

Systems change to define, value and embed lived experience leadership benefits from collective efforts in both formal research and sector development activities. These can be used to generate foundational understandings and guidance for working together in genuine ways for transforming mental health and social sector systems, experience and outcomes.

**Public Contribution:**

Members of lived experience communities codesigned the project, and contributed to project governance and the development of all findings and project reports.

## 1 Introduction

### 1.1 Background

Internationally, there has been a rapid growth in lived experience roles within the mental health and social sectors (WHO 2021 [1, 2, 3]). Predominantly, these roles have been focused on peer worker roles, where people with lived experience come to work in multidisciplinary teams and provide mutual peer support with consumers and carers accessing services [4, 5, 6]. This essential development has occurred alongside other aspects of lived experience leadership at services and systems levels [7]. This includes supporting the growth and influence of lived experience voices in organisational management, service planning, research, policy governance and other places of significant decision‐making impact [8, 9]. These are important places and pathways for people with lived experience to achieve influence and collectively shape systems and services to be recovery‐focused and consumer‐directed. A key issue, from an advocacy perspective, is the need for a clear narrative and resourcing for effectively embedding lived experience leadership, representation, management and governance [5, 10, 11].

International, national and state mental health policy settings mandate lived experience and peer contributions to be made in clinical services, policy development and governance structures [12, 13, 14, 15, 16, 17]. These commitments, however, are often not supported by resource flows in the public health system [18]. The resources that enable robust pathways for developing and training leaders [19], reform of governance processes for more balanced decision making [20] or for enabling coproduction of services as standard practice [21] are not provided in systematic ways. The development of specific and supported pathways is essential, given the ongoing marginalisation and disempowerment that many people with significant mental health and other social issues experience [4]. People with lived experience are more likely to report feeling inferior or experience shame [22, 23], have a sense of powerlessness, lack of control, subordination and social defeat [24]. These experiences impact on the capacity of consumers and carers to have a voice in mental health policy, planning and evaluation. As stigma, marginalisation and disempowerment are historic legacies of social systems and mental models about mental health and illness, there is a need for sustained systems‐level change towards empowerment, inclusion and valuing of lived experience perspectives and personhood.

In wider communities, there remains little understanding and development on the potential of lived experience leadership to share understandings and to shape narratives around mental health issues, suicide prevention and community responses. There is also a need to recognise that lived experience leaders have been pivotal in self‐help, recovery and harm reduction movements, and have a strong history in growing sophisticated consumer and family carer organisations in these spaces [25, 26].

## 2 Objective

This study discusses and reflects on the sector development activities of the Activating Lived Experience Leadership Project (ALEL) as part of a participatory action research (PAR) process for developing an agenda for systems‐level recognition, valuing and embedding of lived experience leadership across the wider mental health and social sector ecosystem in South Australia. We aim to provide details on the project's processes of building a collective partnership among lived experience and other leaders from within the sector, to enable the actions and strategies identified through research to be enacted via sector‐level collaboration. We also describe the collaborative processes used for leaders to agree on eight key action areas for transformative systems change in South Australia. These eight key action areas may be relevant and transferable to other jurisdictions.

### 2.1 Project Background

The ALEL project was a South Australian participatory action research project (2019–2021), which was funded as a research collaboration between LELAN (the South Australian Lived Experience Leadership & Advocacy Network) and the Mental Health and Suicide Prevention Research and Education Group, University of South Australia (UniSA). The project was generously funded through the Fay Fuller Foundation. All positions were designated lived experience roles featuring two lived experience consumer researchers and a lived experience carer researcher. Research approval was obtained from the Human Research Ethics Committee of the University of South Australia in January 2020 (ID 202513).

The project was supported throughout by a project advisory group (PAG) featuring seven lived experience leaders and five other leaders from within the sector, including a mental health service peak body, and government representatives. The participation of the members of the PAG enabled lived experience expertise to advise and guide both community development and formal research activities and strengthened the project as a lived experience–led process. The project also had considerable participation using summit meetings on two occasions to bring together lived experience and other leaders within the sector for integrating codesign methodology and to collectively define high‐level actions for change. The various activities of the projects are described below.

### 2.2 Research Approach

The project used a PAR approach to create a focus on collective discovery, planning, action and reflection [27]. As an approach to research, PAR was chosen as a model of research, which sought to be inclusive and emancipatory, and aligned with a social justice perspective. This enabled a focus on bringing together people with lived experience and other leaders from within the sector to engage in knowledge production to provide transformational change [28].

The research process, in terms of philosophy and design, was centred in PAR methods of collective design, reflection and action (via the research team and PAG members working together). There were also overlaps with user/survivor–led research in the significant closeness between the lived experience researcher identities and the topic of the project, and efforts to include participants as knowledge producers, not subjects of research [29]. The project's epistemology was based on the authority and authenticity of participant perspectives and knowledge either as lived experience of advocacy and leadership or experience of involving or working alongside as told by other sector leaders, and understanding issues and experiences as people expressed them. The research team were self‐aware of the assumptions embedded in the project (lived experience leadership is needed) and how these and the researchers' own experiences could shape design, interpretation and analysis. In terms of design, this included caution about how survivor/user definitions of lived experience framed participation, and potential ‘gate keeping’ in the project [30], and the intentional decision to involve diverse groups of lived experience who may not see mental health as their primary identification as peers.

With a systems focus, the project aimed to shift power dynamics and progress strategic and cultural change across the mental health and social sector ecosystem. This involved the activation and mobilisation of latent resources in our communities to challenge the assumptions and norms of how people in South Australia with mental health issues participate in discussions about services and life needs [31].

The project intentionally used the Water of Systems Change approach [32] as a framework for communicating to stakeholders about key conditions of systems change and how these could be used to interpret the research and consultation findings emerging from the project. The conditions of change in this model locate policies, practices, resource flows, relationships, power dynamics and mental models as sites for attention and action. Using this model heuristically assisted the project team and participants to consider the complex conditions and dynamics that both enable and prevent change across human service systems. It featured in all events and messaging about the project, encouraging a wide focus on systems rather than solely on organisations, and using the model intentionally to consider both visible and invisible dynamics shaping how lived experience leadership is recognised, valued and resourced in the ecosystem. The model assisted the project in designing questions for formal research, understanding the current state of lived experience leadership, interpreting perspectives and experiences offered by participants and guiding many of the conversations across the project.

The design of the project also used a variety of sector development and collective impact strategies [33] to guide action, apart from formal research processes. The separation of formal research activities from sector development was required due to both the scope of the project in systems change and also due to the limits of formal human research ethics on PAR projects, where requirements of anonymity, privacy and ownership of data often constrain processes for mobilising and involving at the community level [34]. This separation and the various activities are described in Table 1. The key processes of engaging and encouraging wider sector involvement were consultations, leaders' summit meetings and the formation of a Communities of Practice.

**Table 1:** Sector development and formal research activities of ALEL.

Sector development activities	Formal research activities
Consultation with leaders (September–October 2019)	Project Advisory Group (governance)
Lived experience leadership group mapping (September–October 2019)	Scoping review (August 2019–July 2020)
Leaders' summit meetings (October 2019 and February 2021) (*n* = 65 participants)	Online focus groups with lived experience leaders (*n* = 31 participants across 11 groups) (April 2020–October 2020)
Using research literature and systems change workshops	Interviews with service and other leaders from within the sector (*n* = 14) (August 2020–October 2020)
Lived experience leadership and change communities of practice	National online survey with lived experience leaders (*n* = 48 respondents) (October 2020–December 2020)
	Thematic analysis and review of themes with the participant advisory group

### 2.3 The Language of Lived Experience Leadership in the Project

The concept of lived experience leadership used in the project needed some definition and qualification, given diverse understandings and histories. Its use in Australia has been influenced by writings of Gordon [35] and O'Hagan [11], who assisted in framing user/survivor/consumer leadership as a paradigm shift away from sole reliance on consumer involvement as service‐based, and limiting ways of understanding user/survivor/consumer action. In these writings, leadership offers a stronger conceptual basis for recognising and understanding influence, advocacy, self‐organisation and management of user/survivor/consumer–run initiatives. In Australia, the term consumer leadership has also been expressed in various states on an industry level, given that consumer is the most commonly used term used in national and state policy. The language of lived experience is, however, also widespread, and generally it is used to refer to personal experience of mental health issues as well as the family/carer experience. Many policy documents and guidelines use lived experience to include both consumer (user/survivor) and family carer, kin perspectives, noting the importance of not conflating these positionalities. These include how peer workforces for each group are located and understood.

This project used this later framing of lived experience to be centred in personal user/survivor/consumer experience but also inclusive of leadership stemming from family/carer experience. This was due to contextual factors such as the existing policy context of (state of project), and close working relationships between consumer and carer peer support specialists on industry levels, indicating that the project would be best likely to succeed by being inclusive of both groups. In using this language and approach, the authors were aware of the very distinct positionings and perspectives of consumers, and carers, and that nearly all of the expressed writings on leadership were from a user/survivor/consumer perspective. We were also aware that leadership as a term may engender distrust from community members, given assumptions of hierarchy and ‘power over’ that can be implied, compared to the values of shared power and equality of many peer mutual aid groups [36]. With these dilemmas, the project team felt the benefits of proceeding overtly with ‘lived experience leadership’ as a paradigm shift was itself an important stance to message to industry and to communities, while also providing space to explore people's feelings and responses to the language within the project's research work.

### 2.4 Collaboration Action in Defining the Current State of Lived Experience Leadership

The project team and PAG provided a central role for planning the sector development and research activities for the project. This occurred with LELAN being the backbone organisation for centring lived experience leadership throughout and beyond the project. Early work involved systems mapping and assessing the current state of lived experience leadership.

This involved consultation with 12 other leaders from both NGO and the government organisations, which enabled us to gather insights and perspectives on identifying conditions that reinforced the current situation and were possible targets of change. It also enabled the team to gather knowledge of projects known to be immersed in this space. The project team also undertook sector‐level mapping to identify areas and groups of lived experience leadership that was not only a part of understanding the current scope of activity but also provided information about networks that could be invited to future project meetings.

Insights from these processes indicated the inconsistent ways of recognising and supporting lived experience leadership in metropolitan, regional and rural areas of South Australia. There were significant examples of partnership, lived experience action, allyship and peer workforce growth occurring across South Australia. However, we observed that successful and impactful work was often dependent on other leaders and managers from within the sector who had ongoing commitments to valuing and embracing lived experience and the hard‐fought gains of individual lived experience leaders. We also observed that progress could easily be lost in the constant change of systems and leaders over time. Members of the project also felt that public health services often engaged leaders in opportunities to meet accreditation standards and that committees mostly operated with low numbers of people with lived experience. Services often failed to provide adequate training, support and mentorship and structure lived experience involvement at the higher levels of decision making. These patterns were more widely felt to occur in regional and rural South Australia, given the lower concentration of resources to enable and support lived experience leadership across vast geographical areas. However, the project was also aware of impressive examples of grass roots lived experience leadership activity, where support group development, suicide prevention projects and policy advocacy had occurred in rural South Australia. The challenges of understanding, valuing, resourcing and embedding lived experience leadership, including recognition of its tremendous potential and claims for justice‐driven change, needed to be developed and implemented at systems levels. This meant improved decision making and resourcing across the ecosystem, providing opportunities for education, training, community building and networking across sectors and embedding lived experience leadership in metropolitan, rural and remote areas so that real and lasting change occurs.

This range of current state information was shared with participants of our first leaders' summit meeting in October 2019 as a process towards collectively identifying shared targets for change at a systems level. This meeting featured PAG members, leaders from mental health statutory and policy units, nongovernment organisations and other community leaders. Leaders at this meeting agreed on future activities for the project, including the need for a Communities of Practice to guide lived experience leadership development, and for creating a framework of understanding for embedding lived experience leadership [37].

### 2.5 Qualitative Research to Define Lived Experience Leadership, Challenges and Pathways for Growth

The project activities described above were part of an iterative PAR approach that incorporated formal qualitative research. This included seven focus groups with 31 participants with a diverse range of lived experience intersectional identities and experiences such as living in rural or regional communities and/or being part of LGBTIQ+ or CALD communities, interviews with other leaders from within the sector (*n* = 14), and an online survey for lived experience leaders in other Australian states (*n* = 48).

Lived experience leader participants were recruited to participate in two rounds of online focus groups (up to 90 min in duration) (face to face was not an option due to COVID‐19 restrictions) to explore the questions of the research project, as well as an online forum (the time commitment for the online forum would be up to 2 h), where they participated anonymously with lived experience participants across the study. A description of the methodology has been reported elsewhere [38].

This process provided data that generated understanding of the roles of lived experience leadership, including the challenges of engaging with services, the effective ways of working with other individuals and groups and the best ways for communicating and having influence. We also sought to understand leaders' perceptions on the systems and organisational strategies for change. Guiding questions were as follows:

- What is the impact of lived experience advocacy and representation on individual recovery from mental health issues, including active participation in broader areas of life?
- What are the reasons why lived experience leadership and advocacy are not well recognised or acknowledged by the health system? What steps are needed to change this?
- What do people with lived experience say is needed in the design of training resources and networks to guide and support lived experience advocacy, representation and leadership?
- How do we ensure that lived experience advocacy, leadership and thinking are embedded in communities and the South Australian mental health ecosystem?

This range of qualitative research was publicly documented and published on an industry level to enable findings to be responded to within the project [39, 40, 41].

This included sharing qualitative findings within PAG meetings (12) and the second leaders' summit meeting. A scoping review on leadership concepts and organisational and systems change was also undertaken, with findings communicated to project stakeholders [42]. The qualitative research findings and scoping review enabled the project to describe the purpose, achievements and potential of lived experience leadership, as a part of participatory action and reflection. Discussion and workshopping during PAG meetings and the second leaders' summit helped the project team to test assumptions, cocreate a vision for change and decide on high‐level strategic actions for improved ecosystem recognition, valuing and embedding of lived experience leadership.

### 2.6 Setting a Vision and Consensus Statement for Systems Transformation

**Figure 1:**
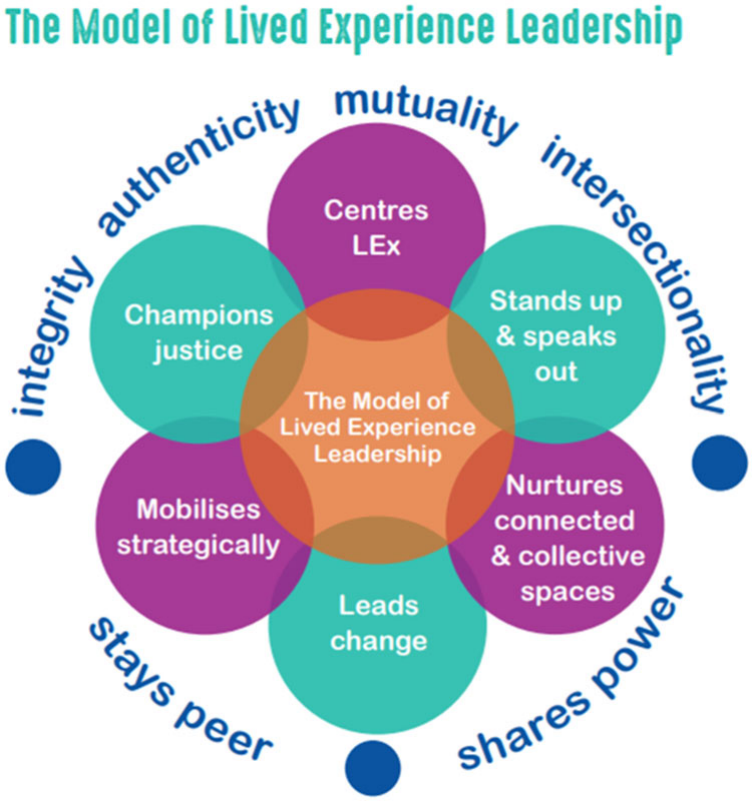
A model for lived experience leadership.

As the project progressed into its second year, PAG discussions planned for three industry‐level documents to help consolidate and express the iterative findings of the project and establish future priorities for change. These included a model of lived experience leadership defining key characteristics, qualities and values [43] (please see Figure 1), a visioning and consensus statement for system transformation [44] and a roadmap‐style document summarising all research findings and priorities for systems transformation [39].

The consensus statement and roadmap were informed by the second leaders' Summit in February 2021, where key findings about the project were presented to leaders for discussion, deliberation and prioritisation [45]. Workshop processes were used to develop a vision for change in South Australia, whereas a consensus process was used to identify the top eight actions for system change from a list of 18 strategies that emerged from the qualitative research activities (please see Table 2). The original 18 strategies were generated from thematic analysis of focus group, interviews and survey data and sent in a briefing to lived experience and other leaders from within the sector attending the meeting. Participants were given eight votes, represented by coloured sticker dots, and asked to place them on their preferred strategies, which were individually listed across venue walls. The consensus setting process discussion among attending leaders also sought to identify key considerations for implementation and ways of working collectively to achieve them. These were subsequently documented within the roadmap.

**Table 2:** Eight actions for systems transformation.

1. Increase the presence of lived experience leaders in governance—designated director positions boards, statutory councils and commissioning groups. 2. Learning and cultural change programmes of lived experience leadership are arranged with executive leaders, staff and communities. 3. Strengthen learning pathways and leadership skills development—advocacy skills, professional development and formal qualifications. 4. Fund leading lived experience organisations to develop and deliver networking activities, coordination of information, events and initiatives that support local lived experience leadership (intersectionality). 5. Enable resource flows for meaningful coproduction of all services and programmes. 6. Promote lived experience leadership and accountability measures through service agreements, KPIs and, where appropriate, regulatory frameworks and legislative processes. 7. Ensure that models of care include equal recognition of lived experience workforces and peer support. 8. Ensure range of organisational and sector infrastructure for the effective recognition, valuing and embedding of the lived experience workforce [44].

### 2.7 Discussion of Key Learnings From ALEL: Using Collective Impact Strategies to Prompt Social Change

This section of the paper reflects on key processes used to organise for collective impact. Table 3 below summarises the ALEL project strategies described in the paper with the five areas of action as defined in Kania and Kramer's model of collective impact (2011). Although the ALEL project was limited in its duration, we believe that these strategies provided an innovative way for lifting the recognition and shared action regarding lived experience leadership among leaders of the state's mental health ecosystem. They also demonstrate an ongoing mechanism for working together beyond the life of the project, and how the eight consensus actions could be realised over the longer term, particularly given the role that LELAN has gained as the recognised peak body for lived experience in South Australia since the project ended.

**Table 3:** ALEL Collective Impact strategies.

Key processes	ALEL strategies
Common agenda	Action research to define leadership, common experiences and required shifts. Building relationships, buy‐in and public will through cross‐sector leadership. Summit meetings to share, develop and confirm agenda and vision for change.
Shared measurement	Research and consultation to identify common understanding of conditions that leaders experience and observe in systems for those with lived experience. Encouraging shared accountability for change.
Mutually reinforcing activities	Encouraging collective and coordinated efforts, rather than organisations working alone. Promoting connections between lived experience and policy leaders, commissioners, services and statutory bodies. Lived experience leadership and change Community of Practice to cultivate leadership skills.
Continuous communication	Newsletters, communications and events to promote goals and achievements of project. Project advisors connected with lived experience networks and policy units, sector peaks. Building trust through messaging, follow‐up and reporting.
Backbone support	LELAN/ALEL as backbone team providing governance, infrastructure and work in other areas as informed by ALEL. Creating and sharing resources by, for and with community and organisations. Network and system mapping to guide strategic planning.

### 2.8 Discussion of ALEL Strategies

This discussion offers a reflection on some of the strategies and processes listed above and the learnings from using action research on sector development and system transformational levels. We raise reflections on the strategies that worked well and commentary on challenges. This is important to share as an increasing number of initiatives, to raise systems‐level recognition of lived experience and to embed lived experience leadership, are occurring across different Australian jurisdictions.

Although there are many system change theories [46], we adopted what was set out by Kania, Kramer and Senge [32]. Systems change in this approach targets multiple levels of action, including policy, practices, resource flows, relationships, power dynamics and conditions that maintain the current state of funding, policy and programme outcomes. These conditions are seen as embedded within cultural mental models that are both explicit and implicit attitudes, assumptions or social norms [32].

### 2.9 Creating a Common (Shared) Agenda

One of the key processes of collective impact was bringing stakeholders together to create a shared agenda for change. In doing this, one of the interesting dynamics was the need to create awareness about lived experience leadership, how it was defined and understood as well as its potential. Although this was part of the formal research process, with a model being produced towards the end of the project, early conversations needed to explore different dimensions and ways of understanding lived experience leadership. This often meant working with diverse, uncertain and incomplete views. Some actors, for instance, saw it as mainly being about consumer and carer involvement in services; others sought clarity on the relationship between lived experience leadership and the peer workforce. There were also conversations about the designated lived experience leader roles in organisations, compared to existing leaders with lived experience, however, not declared publicly. The project's scoping review [42] helped to produce some key understandings and helpful concepts. Furthermore, it also reinforced that lived experience leadership was an emerging idea and challenging to share as both the central subject and the rationale of the project (see also [36]). Over time, the project team used ways to share emerging research findings about how lived experience was being understood by sharing mind maps and other academic papers. Writing the roadmap helped to consolidate a shared understanding by documenting the themes on the characteristic and qualities of lived experience leadership in leading change and also producing a value proposition on what it offered to the wider mental health ecosystem. Our final process was doing the visioning exercise at our second summit meeting, which resulted in the final vision as expressed in the project's Consensus Statement. Both documents have provided foundations for ongoing work to promote understanding and actions to embed lived experience leadership in South Australia.

### 2.10 Engagement for Shared Understanding and Measurement

Another point of reflection is the work of engaging sector leadership, allies and community in and across the project. Early in the project, the project team ran a series of consultation meetings with key leaders from within the sector about their experience, systems change and their vision for it. Part of this was consciously talking about lived experience leadership as a moral imperative and dynamic idea, and inviting leaders who were known to have a commitment or who had significant achievements in supporting the growth of leadership in their organisations and policy projects. Through individual conversations, data were collected that also complemented other mapping work and enabled the team to draft a ‘current state’ assessment of the status and level of lived experience leadership in South Australia. This was then presented at our first summit meeting, where we brought leaders together to workshop further understandings about the current state of lived experience leadership in the South Australian mental health ecosystem. The summit identified and documented them within a framework adapted from the Water for Systems Change model. Assessing the ‘current state’ is a feature of systems change approaches including Collective Impact and enables partners to establish baseline understandings for future measurement of progress [33]. Indicators and outcomes for change were completed for each of the roadmap's eight actions for systems transformation as the first iteration of ways of measuring change.

### 2.11 Mutually Reinforcing Activities

We felt that this process of layering the conversations and engaging leaders both individually and collectively were effective processes for engaging them as partners over time and building the will for change. The first summit demonstrated this will by committing to future actions of convening a community of practice for lived experience leadership, developing a framework for lived experience leadership and holding a follow‐up summit meeting [37], which would play a role as mutually reinforcing activities. Beyond this meeting, LELAN continued to build strategic relationships with other leaders from within the sector and engage them in the project, as well as other LELAN work in engaging policy units and provider organisations in embedding lived experience leadership at governance levels. The success of this was evident in the fact that the second summit workshop, held 13 months later, attracted twice the numbers of leaders.

### 2.12 Continuous Communication

Continuous communication occurred in various informal and formal ways across ALEL. Formally, the project had its research activities, alongside a Communities of Practice, and the activities of the lived experience PAG, which established lines of communication with participants in each group. The project also used periodical newsletters, press releases and high‐profile launches to begin and end the project. Industry‐level research documents reporting on focus groups, interviews and surveys were also made available [39, 40, 41], plus information about events, for example, summit meetings and other networking events. Informally, the project benefited greatly from LELAN's development of networks across lived experience, service and policy leaders, and communication occurring on a daily level to remind about the level of systems change activity.

Although LELAN used its e‐news and website for communication, another way to improve continuous communication could be having a dedicated capability via social media set up for the project. This would enable easier ways to communicate and maintain momentum across the diverse groups and organisations involved in the project. Social media has been growing in use in health education and change projects for over a decade [47, 48]. Although a one‐way digital communication model for this project was helpful, a two‐way communication model (through a forum or a social media) could possibly have created higher engagement for information sharing [48] and provide people an opportunity to respond in their own time. However, we also found that there were considerable barriers to open communication between research activities and the sector‐level events due to research ethical considerations preventing open communication and disclosure by people participating in focus groups and interviews about their role in the project. This was more evident when it came to making connections between their local leadership, and how lived experience leadership was being defined by the project, and what strategies were needed for this to thrive and have impact.

Numerous commentators on PAR have defined the barriers to open participation and knowledge mobilisation that occur due to formal research ethics processes [34, 49]. There were considerable ethical issues to work through for the project team, including understanding and rebalancing power dynamics between the researchers as lived experience and participants, and also safeguarding ethical spaces for participation and voice during summit meetings and other events.

### 2.13 Backbone Support Roles

One of the collective impact processes to reflect on is the need for a backbone organisation. Throughout the project, LELAN and the ALEL partnership provided this role via project governance, convening the PAG, building networks, co‐facilitating a Communities of Practice and in creating resources for community and organisations. These are important backbone functions as understood in the Collective Impact approach [33]. As project funding established the executive director role for 2 years, this enabled continual sector‐level presence in communicating and operationalising strategies for lived experience leadership within the ALEL project, and beyond, in terms of the other project work generated by LELAN. Entering the project, LELAN felt that playing a backbone support role would enable lived experience expertise to be centred as a means of shifting typical power dynamics. This was a key subtext for the project, as building the capability, voice and influence of LELAN as a consumer network would invite established service organisations to engage and seek guidance about embedding lived experience leadership in their decision‐making structures. This role flipped the usual process of consumers and carers only having influence in settings controlled by public health services or NGOs and advocating for involvement. A centring of power for the consumer network enabled a shift in setting agendas for change, who held expertise (e.g., in codesign) and who had capacity to organise and deliver strategic projects. After the project ended, LELAN has continued to grow and consolidate through multiple project grants and has recently received 4 years of continued funding for peak body‐type functions. It continues to play a leadership role in systems‐level change, although the time gap between ALEL funding ending and recent government funding has meant interruptions to the backbone function role during this period. Both partners, LELAN and UniSA, were unable to continue the momentum of ALEL due to limited capacity to organise and bring stakeholders together as per the project. Specific funding for backbone functions and project coordination was fundamental to this level of activity on a state‐wide level.

## 3 Strengths and Limitations

The project's strengths include being led by lived experienced researchers plus being guided by the knowledge and wisdom of the lived experience PAG, using a PAR approach and various coordinated research and sector development activities. A strength also lay in efforts to promote intersectionality and the diversity of project participants. There is no formal evaluation of the activities and reach of the ALEL project in creating lasting change on a systems level in South Australia. The activities and processes described in the paper should be seen as sharing of project experience for early‐stage systems change work in engagement, establishing a shared agenda, assessing the current state of the issue in a systems perspective and creating a plan for change. There may be divergent viewpoints on the effectiveness of these strategies as experienced by participants and partner organisations of the project.

## 4 Conclusion

This study has presented a research team's perspective on a systems change project: *Advancing Lived Experience Leadership*. Key strategies, processes and challenges are discussed for working collectively within a PAR methodology. The story behind creating the *Roadmap for strengthening lived experienced leadership for transformative systems change in South Australia* provides an inside view of working together within a lived experience and allyship framework, including guidance, processes and strategies, for working together in genuine ways for transforming mental health systems, experience and outcomes.

## Author Contributions

**Mark Loughhead:** conceptualisation, methodology, data curation, investigation, validation, formal analysis, supervision, funding acquisition, writing–original draft, writing–review and editing. **Ellie Hodges:** conceptualisation, methodology, investigation, validation, formal analysis, supervision, funding acquisition, project administration, writing–original draft, writing–review and editing. **Heather McIntyre:** methodology, data curation, investigation, formal analysis, project administration, writing–original draft, writing–review and editing. **Nicholas Procter:** conceptualisation, methodology, funding acquisition, investigation, supervision, writing–review and editing. **Anne Barbara:** validation, writing–review and editing, methodology. **Brooke Bickley:** methodology, validation, writing–review and editing. **Lee Martinez:** methodology, validation, writing–review and editing. **Leticia Albrecht:** methodology, validation, writing–review and editing. **Lisa Huber:** methodology, validation, writing–review and editing.

## Ethics Statement

This research was carried out in accordance with the Declaration of Helsinki and was approved by the University of South Australia Human Research Ethics Committee (Reference: 202513).

## Conflicts of Interest

The authors declare no conflicts of interest.

## Data Availability

Due to ethics restrictions, qualitative data will not be available.

## References

[1] M. Crowe and L. Sheppard , “Scoping Review of Health Outcomes for People With Disabilities in User‐Led Organisations,” Australian Journal of Primary Health 27, no. 5 (2021): 339–349. 34649643 10.1071/PY20193

[2] N. Jones , K. Atterbury , L. Byrne , M. Carras , M. Brown , and P. Phalen , “Lived Experience, Research Leadership, and the Transformation of Mental Health Services: Building a Researcher Pipeline,” Psychiatric Services 72, no. 5 (2021): 591–593. 33691492 10.1176/appi.ps.202000468

[3] N. Jones , G. B. Teague , J. Wolf , and C. Rosen , “Organizational Climate and Support Among Peer Specialists Working in Peer‐Run, Hybrid and Conventional Mental Health Settings,” Administration and Policy in Mental Health and Mental Health Services Research 47, no. 1 (2020): 150–167. 31564032 10.1007/s10488-019-00980-9

[4] L. Byrne , C. Roper , B. Happell , and K. Reid‐Searl , “The Stigma of Identifying as Having a Lived Experience Runs Before Me: Challenges for Lived Experience Roles,” Journal of Mental Health 28, no. 3 (2019): 260–266. 27841058 10.1080/09638237.2016.1244715

[5] L. Byrne , A. Stratford , and L. Davidson , “The Global Need for Lived Experience Leadership,” Psychiatric Rehabilitation Journal 41, no. 1 (2018): 76–79. 29494198 10.1037/prj0000289

[6] L. Byrne , H. Roennfeldt , Y. Wang , and P. O'Shea , “‘You Don't Know What You Don't Know’: The Essential Role of Management Exposure, Understanding and Commitment in Peer Workforce Development,” International Journal of Mental Health Nursing 28, no. 2 (2019): 572–581. 30609234 10.1111/inm.12562

[7] L. Byrne , H. Roennfeldt , J. Wolf , et al., “Effective Peer Employment Within Multidisciplinary Organizations: Model for Best Practice,” Administration and Policy in Mental Health and Mental Health Services Research 49, no. 2 (2022): 283–297. 34478040 10.1007/s10488-021-01162-2

[8] S. Stewart , B. Scholz , S. Gordon , and B. Happell , “‘It Depends What You Mean By Leadership’: An Analysis of Stakeholder Perspectives on Consumer Leadership,” International Journal of Mental Health Nursing 28, no. 1 (2019): 339–350. 30281898 10.1111/inm.12542

[9] L. Byrne , L. Wang , H. Roennfeldt , et al., National Lived Experience Workforce Guidelines (Canberra: National Mental Health Commission, 2021).

[10] E. Hodges , A. Leditschke , and L. Solonsch , The Lived Experience Governance Framework: Centring People, Identity and Human Rights for the Benefit of All, SA Lived Experience Leadership & Advocacy Network, for the National Mental Health Consumer and Carer Forum and the National PHN Mental Health Lived Experience Engagement Network (Canberra: Mental Health Australia, 2023), https://www.lelan.org.au/wp‐content/uploads/2023/08/Lived‐Experience‐Governance‐Framework.pdf.

[11] M. O'Hagan , “Leadership for Empowerment and Equality: A Proposed Model for Mental Health User/Survivor Leadership,” Journal of Leadership in Public Services 5, no. 4 (2009): 1–13.

[12] K. Baker , M. Ferguson , M. Loughhead , and N. G. Procter , Mental Health Safety and Quality Engagement Guide (Australia: National Mental Health Commission, 2020).

[13] National Health and Medical Research Council, Australian Research Council and Universities Australia , National Statement on Ethical Conduct in Human Research (Canberra: National Health and Medical Research Council, 2023).

[14] National Mental Health Commission , Monitoring Mental Health and Suicide Prevention Reform: National Report (Sydney: National Mental Health Commission, 2018).

[15] SA Health , Mental Health Services Plan 2020‐2025 (Adelaide: SA Health, 2019), https://www.sahealth.sa.gov.au/wps/wcm/connect/8520124e‐0250‐4393‐819e‐71bca0db4ad9/19032.2+MHSP‐report‐web‐no+watermark.pdf?MOD=AJPERES&CACHEID=ROOTWORKSPACE‐8520124e‐0250‐4393‐819e‐71bca0db4ad9‐nwLp6cp.

[16] South Australian Mental Health Commission , South Australian Mental Health Strategic Plan 2017–2022 (Adelaide: SA Health and Wellbeing, 2017).

[17] World Health Organization , Comprehensive Mental Health Action Plan 2013‐2030 (Geneva: World Health Organization, 2021), https://iris.who.int/bitstream/handle/10665/345301/9789240031029‐eng.pdf.

[18] R. White , A. Wells , and H. Hammou , *Funding Grassroots Mental Health Work: How Funders Can Better Resource User‐Led Groups Working to Support the Mental Health of Their Communities* (London: National Survivor User Network, 2022), https://www.nsun.org.uk/resource/funding‐grassroots‐mental‐health‐work/.

[19] A. Newton , A. Beales , D. A. Collins , and T. Basset , “Service User Leadership: Training and Development for Service Users to Take the Lead,” The Journal of Mental Health Training, Education and Practice 8, no. 3 (2013): 134–140.

[20] B. Scholz , J. Bocking , and B. Happell , “Breaking Through the Glass Ceiling: Consumers in Mental Health Organisations' Hierarchies,” Issues in Mental Health Nursing 38, no. 5 (2017): 374–380, 10.1080/01612840.2017.1280106. 28448229

[21] C. Roper , F. Grey , and E. Cadogan , Co‐Production: Putting Principles Into Practice in Mental Health Contexts (Melbourne: University of Melbourne, 2018).

[22] S. L. Johnson , L. J. Leedom , and L. Muhtadie , “The Dominance Behavioral System and Psychopathology: Evidence From Self‐Report, Observational, and Biological Studies,” Psychological Bulletin 138, no. 4 (2012): 692–743. 22506751 10.1037/a0027503 PMC3383914

[23] H. Roennfeldt , M. Wyder , L. Byrne , N. Hill , R. Randall , and B. Hamilton , “Subjective Experiences of Mental Health Crisis Care in Emergency Departments: A Narrative Review of the Qualitative Literature,” International Journal of Environmental Research and Public Health 18, no. 18 (2021): 9650. 34574574 10.3390/ijerph18189650 PMC8471743

[24] R. Wilkinson and K. Pickett , The Inner Level: How More Equal Societies Reduce Stress, Restore Sanity and Improve Everyone's Well‐Being (London: Penguin Books, 2019). 10.3399/bjgp19X705377 PMC6715479 31467020

[25] G. Brown , D. Reeders , A. Cogle , et al., “Tackling Structural Stigma: A Systems Perspective,” supplement, *Journal of the International AIDS Society* 25, no. Suppl 1: e25924. 10.1002/jia2.25924 PMC9274342 35818874

[26] L. D. Brown and S. Rogers , “The Impact of Mental Health Consumer‐Run Organizations on Transformative Change,” in Community Psychology and Community Mental Health: Towards Transformative Change, eds. G. Nelson , B. Kloos , and J. Ornelas . (Oxford Academic, New York, 2014), 10.1093/acprof:oso/9780199362424.003.0006.

[27] F. Baum , “Participatory Action Research,” Journal of Epidemiology & Community Health 60, no. 10 (2006): 854–857. 16973531 10.1136/jech.2004.028662 PMC2566051

[28] T. E. Benjamin‐Thomas , A. M. Corrado , C. McGrath , D. L. Rudman , and C. Hand , “Working Towards the Promise of Participatory Action Research: Learning From Ageing Research Exemplars,” International Journal of Qualitative Methods 17, no. 1 (2018): 160940691881795.

[29] J. Russo , “Survivor‐Controlled Research: A New Foundation for Thinking About Psychiatry and Mental Health,” Forum Qualitative Sozialforschung/Forum: Qualitative Social Research 13, no. 1 (2012): 1–29.

[30] D. Rose , “Participatory Research: Real or Imagined,” Social Psychiatry and Psychiatric Epidemiology 53 (2018): 765–771. 29931442 10.1007/s00127-018-1549-3 PMC6061456

[31] C. Curtis , I. Burkett , and C. Vanstone , Examples and Emerging Insights From TACSI's Big Change Work. What We're Learning About Systems Change Through Practice (Adelaide: The Australian Centre for Social Innovation, 2018).

[32] J. Kania , M. Kramer , and P. Senge , The Water of Systems Change (Washington DC: FSG, 2018).

[33] J. Kania and M. Kramer , “Collective Impact,” Stanford Social Innovation Review 9, no. 1 (2011): 36–41, 10.48558/5900-KN19.

[34] S. Banks , A. Armstrong , K. Carter , et al., “Everyday Ethics in Community‐Based Participatory Research,” Contemporary Social Science 8, no. 3 (2013): 263–277, 10.1080/21582041.2013.769618.

[35] S. Gordon , “The Role of the Consumer in the Leadership and Management of Mental Health Services,” Australasian Psychiatry 13, no. 4 (2005): 362–365. 16403131 10.1080/j.1440-1665.2005.02215.x

[36] R. Waddingham , Lived Experience Leadership—Mapping the Lived Experience Landscape in Mental Health (2021), https://www.nsun.org.uk/wp‐content/uploads/2021/11/LEL‐Report‐Nov‐2021.pdf.

[37] E. Hodges , M. Loughhead , H. McIntyre , and N. G. Procter , System & Sector Leaders' Summit: Dialoguing for Change (Adelaide: SA Lived Experience Leadership and Advocacy Network and University of South Australia, 2019), www.lelan.org.au/alel.

[38] M. Loughhead , E. Hodges , H. McIntyre , et al., “A Model of Lived Experience Leadership for Transformative Systems Change: Activating Lived Experience Leadership (ALEL) Project,” Leadership in Health Services 36, no. 1 (2023): 9–23. 10.1108/LHS-04-2022-0045 35943397

[39] M. Loughhead , E. Hodges , H. McIntyre , and N. G. Procter , A Roadmap for Strengthening Lived Experience Leadership for Transformative Systems Change in South Australia (Adelaide: SA Lived Experience Leadership and Advocacy Network and University of South Australia, 2021), www.lelan.org.au/alel.

[40] M. Loughhead , E. Hodges , H. McIntyre , and N. Procter , Activating Lived Experience Leadership: Summary Report on Focus Group Research With Lived Experience Leaders (Adelaide: University of South Australia and the Lived Experience Advocacy and Leadership Network SA, 2021), www.lelan.org.au/alel.

[41] M. Loughhead , E. Hodges , H. McIntyre , and N. Procter , Activating Lived Experience Leadership: Summary Report on Research Interviews With Sector and Service Leaders (Adelaide: University of South Australia and the Lived Experience Advocacy and Leadership Network SA, 2021), www.lelan.org.au/alel.

[42] M. Loughhead , H. McIntyre , E. Hodges , and N. G. Procter , Lived Experience Leadership for Organisational and Systems Change: A Scoping Review of Concepts and Evidence (Adelaide: University of South Australia and Lived Experience Leadership and Advocacy Network SA, 2020).

[43] E. Hodges , M. Loughhead , H. McIntyre , and N. G. Procter , The Model of Lived Experience Leadership (Adelaide: Lived Experience Leadership and Advocacy Network and University of South Australia, 2021), www.lelan.org.au/alel.

[44] E. Hodges , M. Loughhead , H. McIntyre , and N. G. Procter , Strengthening Lived Experience Leadership for Transformative Systems Change: A South Australian Consensus Statement (Adelaide: SA Lived Experience Leadership and Advocacy Network and University of South Australia, 2021), www.lelan.org.au/alel.

[45] E. Hodges , M. Loughhead , H. McIntyre , and N. G. Procter , System & Sector Leaders’ Summit: Dialoguing for Change (Adelaide: SA Lived Experience Leadership and Advocacy Network and University of South Australia, 2021), www.lelan.org.au/alel.

[46] J. S. Walker , N. Koroloff , and S. J. Mehess , “Community and State Systems Change Associated With the Healthy Transitions Initiative,” The Journal of Behavioral Health Services & Research 42, no. 2 (2015): 254–271. 25537434 10.1007/s11414-014-9452-5

[47] S. Fox and K. Purcell , Social Media and Health (Washington, DC: R. Pew Research Centre, 2010), https://www.pewresearch.org/internet/2010/03/24/social‐media‐and‐health/.

[48] L. Walsh , N. Hyett , N. Juniper , C. Li , S. Rodier , and S. Hill , “The Use of Social Media as a Tool for Stakeholder Engagement in Health Service Design and Quality Improvement: A Scoping Review,” Digital Health 7 (2021): 205520762199687, 10.1177/2055207621996870. PMC7917429 33717499

[49] N. Khanlou and E. Peter , “Participatory Action Research: Considerations for Ethical Review,” Social Science & Medicine (1982) 60, no. 10 (2005): 2333–2340. 15748680 10.1016/j.socscimed.2004.10.004

